# A microgenetic approach to the relationship between creativity and aggression in mental disorders

**DOI:** 10.1192/j.eurpsy.2021.2077

**Published:** 2021-08-13

**Authors:** H. Yaniv

**Affiliations:** Criminology, Bar Ilan University, Ramat Gan, Israel

**Keywords:** Creativity, Aggression, microgenetic method, sublimation - defense mechanism

## Abstract

**Introduction:**

A study, examined creativity and aggression in individuals suffering from mental disorders, will present.

**Objectives:**

Five groups: individuals suffering from mental disorders – with or without a history of aggression; creative individuals; aggressive individuals; and a control group

**Methods:**

Different personality questionnaires: anxiety, absorption, schizotypy and humor and the microgenetic method – a projection technique, using visual stimulation by means of a computerized experiment. The stimuli that were presented in this study were two artworks. They were presented in a blurry manner that became clearer and the exposure time became longer.

**Results:**

An emotional process aroused, when exposed to the stimuli. The stimuli had an influence on the subject’s internal world, which was reflected back in the verbal reports, especially the aspects of creativity and aggression. Secondly, the pathological groups were the lowest in the expression of creativity. However, they expressed more aggressive expression than expected while exposed to the stimuli. Moreover, these groups were ranked high, in traits related to creativity, such as schizotypy, absorption and the P dimension.

**Conclusions:**

The Microgenetic-method gave a better perspective of the participant’s personality and can be used as a diagnostic-tool. In addition, the research demonstrates that creativity and aggression are multi-dimensional traits and are noticeably present in the pathological groups. Therefore, a person coping with psychopathology should get assistance with controlling the expression of his urges. They should be directed towards a creative expression of their inner-world, allowing a full range of expression of his strengths and pains. This will sublimate the feelings of aggression.

**Disclosure:**

No significant relationships.

